
JOURNAL INFORMATION
==============================
NLM Title Abbreviation: Function (Oxf)
Journal ID: Function (Oxf)
NLM Journal ID: 101770668
Journal ID: function

Function (Oxford, England)

EISSN: 2633-8823

ARTICLE INFORMATION
==============================
PMCID: PMC7313979
PMID: 38626245
DOI: 10.1093/function/zqaa003
Article ID: zqaa003
Article version: 1
Subjects: Perspectives

Endocytic uptake of SARS-CoV-2: the critical roles of pH, Ca2+ and NAADP

Petersen Ole H PetersenOH@cardiff.ac.uk
Gerasimenko Oleg V
Gerasimenko Julia V
School of Biosciences, Cardiff University , Wales, UK
Correspondence: Ole H Petersen, School of Biosciences, Cardiff University, Sir Martin Evans building, Museum Avenue, Cardiff CF10 3AX, Wales, UK. Email: PetersenOH@cardiff.ac.uk
Electronic publication date: 2020 Jun 5
Electronic Location ID: zqaa003
Received 2020 May 26; Revised 2020 Jun 1; Accepted 2020 Jun 2
Copyright: © American Physiological Society 2020.
Copyright year: 2020
License: This is an Open Access article distributed under the terms of the Creative Commons Attribution License (http://creativecommons.org/licenses/by/4.0/), which permits unrestricted reuse, distribution, and reproduction in any medium, provided the original work is properly cited.
License URL: https://creativecommons.org/licenses/by/4.0/

Page count: 7
edited-state: accepted-manuscript
article-lifecycle: PAP

==============================
*Perspective*

### Endocytic uptake of SARS-CoV-2: the critical roles of pH, $Ca^{2+}$ and NAADP

**Ole H Petersen, Oleg V Gerasimenko & Julia V Gerasimenko**

**School of Biosciences, Cardiff University, Wales, UK**

Correspondence: Ole H Petersen
School of Biosciences
Cardiff University
Sir Martin Evans building
Museum Avenue
Cardiff CF10 3AX, Wales, UK
Email: PetersenOH@cardiff.ac.uk

© The Author(s) 2020. Published by Oxford University Press on behalf of the American Physiological Society.

This is an Open Access article distributed under the terms of the Creative Commons Attribution License (http://creativecommons.org/licenses/by/4.0/), which permits unrestricted reuse, distribution, and reproduction in any medium, provided the original work is properly cited.

It has recently been shown that SARS-CoV-2 enters cells via receptor-mediated endocytosis, as expected from previous studies of other coronaviruses [1]. ACE2 is the receptor for SARS-CoV-2, which unlocks access to the endocytic uptake of the virus [2]. It has also been demonstrated that a two-pore channel (TPC2) is critically important for SARS-CoV-2 entry into cells [1]. From earlier studies we know that TPCs are channels in the endo-lysosomal system through which $Ca^{2+}$ is released when activated by the intracellular messenger nicotinic acid adenine dinucleotide phosphate (NAADP) [3]. The new findings on SARS-CoV-2 entry [1] link two hitherto completely separate research fields, namely physiological $Ca^{2+}$ signaling and virology, opening up intriguing and important opportunities for future work on the cellular patho-physiology of COVID-19.

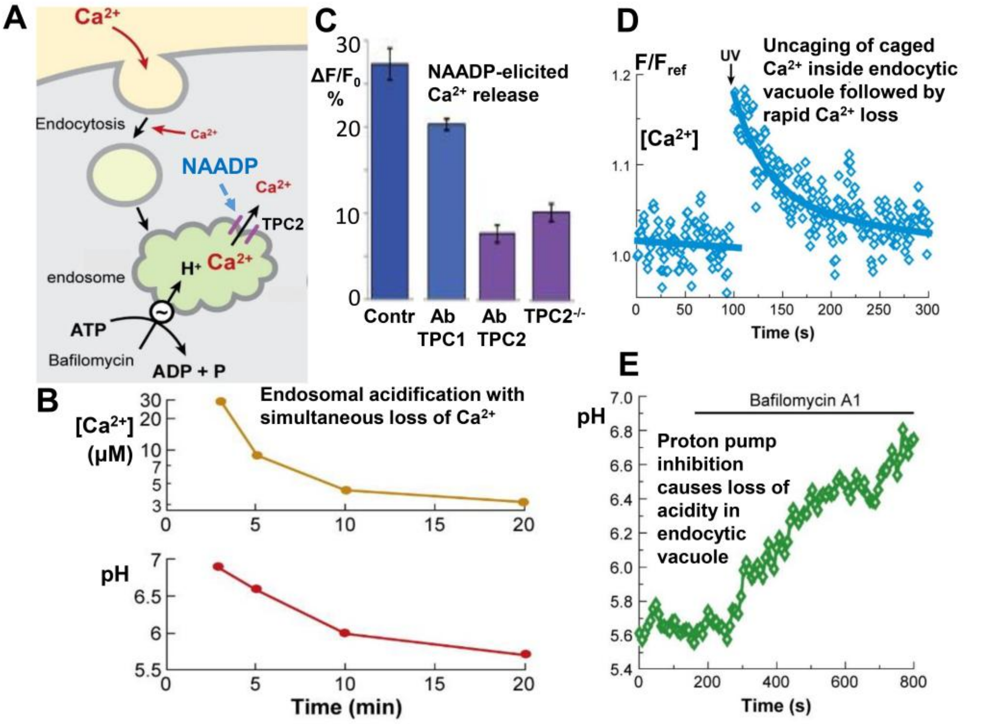

*Fig. 1. The link between endosomal acidification and loss of $Ca^{2+}$ from endosomal organelles.* ***A*** *Simplified sketch of endosomal $Ca^{2+}$ uptake, acidification of the endosome by the bafilomycin-sensitive proton pump and leak of $Ca^{2+}$ from the endosome, via NAADP-sensitive TPCs.* ***B*** *Time course of endosomal acidification and $Ca^{2+}$ loss in fibroblasts.* ***C*** *Effects of antibodies against TPC1 and TPC2 as well as deletion of TPC2 on NAADP-elicited $Ca^{2+}$ release from permeabilized pancreatic acinar cells.* ***D*** *Rapid loss of $Ca^{2+}$ from pancreatic acinar endocytic vacuole following sudden elevation of [$Ca^{2+}$] inside vacuole due to $Ca^{2+}$ uncaging.* ***E*** *Rapid increase in pH of pancreatic acinar endocytic vacuole after blockage of proton pump by 100 nM bafilomycin A1. Data in B are from Gerasimenko et al 1998 [4], in C from Gerasimenko et al 2015 [5] and in D and E from Sherwood et al 2007 [6]*

Endocytosis is a process whereby substances in the extracellular fluid can be taken into cells without endangering the integrity of the plasma membrane (Fig. 1). Endosomes have an acid interior due to the operation of a proton pump that can be very specifically inhibited by bafilomycin (Fig. 1). Ou et al al [1] have shown that SARS-CoV-2 entry is completely blocked when the proton pump is inhibited by 100 nM bafilomycin A, a concentration of the inhibitor that has previously been shown to prevent endosomal acidification in different cell types (Fig. 1) [4,6].

It is particularly intriguing that pharmacological inhibition of TPC2, with tetrandrine, markedly reduced cellular entry of SARS-CoV-2 [1]. This finding is similar to what has been shown in much more detail with regard to the entry of the Ebola virus [7]. Deletion of TPC1 or TPC2 resulted in marked inhibition of virus entry [7]. Furthermore, several known inhibitors of NAADP-elicited $Ca^{2+}$ release from acid stores, for example, Ned-19 and tetrandrine, also severely reduced entry of this virus [7]. From earlier $Ca^{2+}$ signaling studies, it is known that deletion of TPC2 markedly reduced NAADP-elicited $Ca^{2+}$ release in permeabilized cells, as did an antibody to TPC2 (Fig. 1). $Ca^{2+}$ release mediated by NAADP in intact cells was also markedly inhibited by Ned-19 [5].

Endocytic acidification occurs at the same time as $Ca^{2+}$ is lost from endosomes (Fig. 1). The fluid taken into cells by endocytosis has, of course, the normal concentration of free $Ca^{2+}$ found in the extracellular fluid, namely ~1mM. However, as seen in Fig. 1, at the earliest time points allowing assessment of endosomal [$Ca^{2+}$], the concentration had already dropped to ~30 μM, which is still markedly higher than the level in the cytosol (~0.1 μM). The endosomal [$Ca^{2+}$] then declined further to ~4 μM in late endosomes (Fig. 1). Endosomal acidification and loss of $Ca^{2+}$ are interlinked. Blockade of the proton pump by bafilomycin prevented release of $Ca^{2+}$ from the endosomes, with endosomal [$Ca^{2+}$] remaining high [4]. If [$Ca^{2+}$] in the early endosomes was kept low, by reducing the extracellular [$Ca^{2+}$] to less than 300 μM, acidification did not occur [4]. The mechanism underlying the linkage between $Ca^{2+}$ and $H^+$ movements is not fully understood, but the fact that the uptake of $H^+$ into endosomes occurs simultaneously with release of $Ca^{2+}$, and that the two processes are interdependent, means that it is difficult to determine whether inhibition of virus entry by blockade of the proton pump or by TPC inhibition [1,7] is primarily due to the high pH or to the relatively high [$Ca^{2+}$] in the endosomes, or to a mixture of both.
Since TPCs are NAADP-sensitive channels, it is important to understand the mechanism by which NAADP is formed. The CD38 enzyme responsible for NAADP generation is an ecto-enzyme, that is internalized by endocytosis [8], and requires an acid environment to perform its task [8]. In pancreatic acinar cells, the process of NAADP formation and the mechanism of action of this messenger have been investigated in some detail [5,8]. In this cell type there is evidence showing that it is in the acidic endosomes that NAADP generation takes place [8]. Furthermore, the initial $Ca^{2+}$ release triggered by NAADP may depend on actions of this messenger on both endosomes and lysosomes [5]. At least part of the effect of proton pump inhibition on virus entry [1,7] could therefore be explained by lack of NAADP formation [8] and action, thereby preventing TPC activation [5].

SARS-CoV-2 is not only entering cells in the respiratory tract [2], but is also taken up in the central nervous system, where it can cause serious damage [9], as well as in the gastro-intestinal tract, including the pancreas [10]. From a mechanistic perspective, it may be of particular interest to study virus uptake in pancreatic acinar cells, since in these cells there is clear evidence for a physiological role of NAADP [5]. In this context it is interesting that COVID-19 can be associated with acute pancreatitis and that ACE2 is present in the pancreas [10]. In acute pancreatitis, a human disease in which the pancreas digests itself, there is a secretion defect and large endocytic vacuoles, which are different from endosomes and post-exocytic in nature, appear in the acinar cells [6]. These vacuoles nevertheless have functional features in common with endosomes. When NAADP is generated by hormonal stimulation, the vacuole membrane is very permeable to $Ca^{2+}$, as demonstrated by the rapid loss of $Ca^{2+}$ from vacuoles following a sudden increase in [$Ca^{2+}$] elicited by uncaging of caged $Ca^{2+}$ by UV light (Fig. 1D). The endocytic vacuoles are also acidic, with a pH similar to that found in endosomes, and bafilomycin A1 - the very specific proton pump blocker - rapidly increased pH inside the vacuoles (Fig. 1E). The endocytic vacuoles may provide an additional and important entry pathway for viruses, including SARS-CoV-2. Furthermore, the post-exocytic vacuoles contain large amounts of many different proteases [6], which could also play a role in SARS-CoV-2 infection.

Exocrine pancreatic secretion is principally controlled by the neurotransmitter acetylcholine (ACh) and the hormone cholecystokinin (CCK). Both agents elicit secretion by mobilising intracellular $Ca^{2+}$, which subsequently triggers $Ca^{2+}$ entry from the extracellular solution. CCK primarily evokes $Ca^{2+}$ release mediated by NAADP acting on TPCs - but also on ryanodine receptors - in a complex arrangement involving endosomes and lysosomes [5]. In contrast, ACh primarily causes intracellular $Ca^{2+}$ release mediated by inositol trisphosphate ($IP_3$), acting on $IP_3$ receptors principally located in the endoplasmic reticulum [5]. Given that endosomal acidification depends on endosomal $Ca^{2+}$ release [4], and that the entry of SARS-CoV-2 and Ebola virus is abolished or severely reduced when the proton pump is blocked or when TPCs are inhibited [1,7], it would be of considerable interest to know if and how NAADP-dependent $Ca^{2+}$ signalling influences SARS-CoV-2 entry. Taking advantage of the co-existence in pancreatic acinar cells of two different $Ca^{2+}$ signaling mechanisms, activated by separate receptor pathways, it would seem possible to provide well defined test and control conditions in such studies.

We conclude that there is evidence for the involvement of acidic endosomes and TPC-mediated $Ca^{2+}$ release from the endo-lysosomal system in both SARS-CoV-2 entry and NAADP-mediated $Ca^{2+}$ signaling. The pancreatic acinar cells may prove to be a particularly useful system for exploring the relationship between these phenomena.

**Conflicts of Interest:** Authors declare no conflicts of interest.

**Funding acknowledgements:** Experimental work in the authors' laboratory was supported by grants from the UK's Medical Research Council (MR/J002771/1 and G19/22/2).

### References

[**1**] Ou X, Liu Y, Lei X, Li P, Mi D, Ren L, Guo L, Guo R, Chen T, Hu J, Xiang Z, Mu Z, Chen X, Chen J, Hu K, Jin Q, Wang J and Qian Z. Characterization of spike glycoprotein of SARS-CoV-2 on virus entry and its immune cross-reactivity with SARS-CoV. *Nature Communications* **11**, 1620, 2020.

[**2**] Hoffmann M, Kleine-Weber H, Schroeder S, Krüger N, Herrler T, Erichsen S, Schiergens TS, Herrler G, Wu N-H, Nitsche A, Müller MA, Drosten C and Pöhlmann S. SARS-CoV-2 cell entry depends on ACE2 and TMPRSS2 and is blocked by a clinically proven protease inhibitor. *Cell* **181**, 271-280, 2020.

[**3**] Calcraft PJ, Ruas M, Pan Z, Cheng X, Arredouani A, Hao X, Tang J, Rietdorf K, Teboul L, Chuang K-T, Lin P, Xiao R, Wang C, Zhu Y, Lin Y, Wyatt CN, Parrington J, Ma J, Evans M, Galione A and Zhu MX. NAADP mobilizes calcium from acidic organelles through two-pore channels. *Nature* **459**, 596-600, 2009.

[**4**] Gerasimenko JV, Tepikin AV, Petersen OH and Gerasimenko OV. Calcium uptake via endocytosis with rapid release from acidifying endosomes. *Current Biology* **8**, 1335-1338, 1998.

[**5**] Gerasimenko JV, Charlesworth RM, Sherwood MW, Ferdek PE, Mikoshiba K, Parrington J, Petersen OH and Gerasimenko OV. Both RyRs and TPCs are required for NAADP-induced intracellular $Ca^{2+}$ release. *Cell Calcium* **58**, 237-245, 2015.

[**6**] Sherwood MW, Prior I, Voronina SG, Barrow SL, Woodsmith JD, Gerasimenko OV, Petersen OH and Tepikin AV. Activation of trypsinogen in large endocytic vacuoles of pancreatic acinar cells. *Proc Natl Acad Sci* (*PNAS*) 104, 5674-5679, 2007.

[**7**] Sakurai Y, Kolokoltsov AA, Chen C-C, Tidwell MW, Bauta WE, Klugbauer N, Grimm C, Wahl-Schott C, Biel M and Davey RA. Two-pore channels control Ebola virus host cell entry and are drug targets for disease treatment. *Science* **347**, 995-998, 2015.

[**8**] Cosker F, Cheviron N, Yamasaki M, Menteyne A, Lund FE, Moutin M-J, Galione A and Cancela J-M. The ecto-enzyme CD38 is a nicotinic acid adenine dinucleotide phosphate (NAADP) synthase that couples receptor activation to $Ca^{2+}$ mobilization from lysosomes in pancreatic acinar cells. *J Biol Chem* **285**, 38251-38259, 2010.

[**9**] Steardo L, Steardo L Jr, Zorec R and Verkhratsky A. Neuroinfection may contribute to pathophysiology and clinical manifestations of COVID-19. *Acta Physiologica* in press, 2020 Doi: 10.1111/apha.13473

[**10**] Hadi A, Werge M, Kristiansen KT, Pedersen UG, Karstensen JG, Novovic S and Gluud LL. Coronavirus Disease-19 (COVID-19) associated with severe acute pancreatitis: Case report on three family members. *Pancreatology* in press, 2020
Doi: 10.1016/j.pan.2020.04.021
